# Diagnostic performance of the QIAstat-Dx gastrointestinal panel compared with conventional methods in pediatric gastroenteritis

**DOI:** 10.1128/spectrum.00055-26

**Published:** 2026-05-19

**Authors:** Neama Salieh, Andrés Pérez-López, Muhammad Iqbal, Aya Abdelal, Mohammed Suleiman

**Affiliations:** 1 Department of Pathology, Sidra Medicine, Doha, Qatar; 2 Department of Pathology and Laboratory Medicine, Weill Cornell Medicine in Qatar 36579, Doha, Qatar; 3 Department of Biomedical Sciences, Qatar University 61780, Doha, Qatar; The George Washington University School of Medicine and Health Sciences, Washington, DC, USA

**Keywords:** pediatric gastroenteritis, infection, multiplex PCR, turnaround time

## Abstract

Pediatric gastroenteritis remains a significant public health issue, underscoring the need for better laboratory diagnostic methods. Traditional diagnostic techniques, such as stool culture and ova and parasite (O&P) examination, are limited by long turnaround times and low detection rates. This study assessed the diagnostic capabilities of the QIAstat-Dx Gastrointestinal Panel (QGP), a multiplex PCR test, against conventional stool culture and O&P examination in 4,801 clinical stool samples from pediatric patients at Sidra Medicine. The study involved stool specimens collected from patients aged ≤18 years who were being tested for gastroenteritis using one or more of the following methods: QGP, stool culture, and O&P examination. QGP demonstrated a higher pathogen detection rate (42.3%; 2,033/4,801) than stool culture (12.6%; 255/2,018) and O&P examination (6.3%; 63/986). A total of 2,814 pathogens were identified in the 2,033 QGP-positive samples, with EPEC being the most prevalent pathogen found in 631 samples (13.1%). QGP showed high concordance with conventional methods, with >98% negative percent agreement (NPA) for all targets and 100% positive percent agreement (PPA) for all targets, except *Salmonella* spp. (84.4% PPA). The lower PPA for *Salmonella* spp. was attributed to isolates detected only by culture after enrichment with Selenite F broth. QGP had a shorter median turnaround time from sample collection to final results (2.8 h) than stool culture (67.6 h) and O&P examination (38.8 h). QGP is a valuable diagnostic tool due to improved detection rate, ability to detect co-infection, and quicker turnaround time, providing a more efficient and comprehensive approach in pediatric gastroenteritis diagnosis.

**IMPORTANCE:**

This study highlights the benefits of using multiplex PCR panels, such as the QIAstat-Dx Gastrointestinal Panel (QGP), which provides results for 23 pathogens simultaneously within 80 min. This extensive retrospective study evaluated the performance of QGP in 4,801 stool samples collected in a pediatric tertiary care center in Qatar. The QGP showed superior diagnostic performance compared with traditional methods, including enhanced pathogen detection and faster results, helping clinicians optimize the treatment of children with gastroenteritis.

## INTRODUCTION

Acute gastroenteritis is the third leading cause of death among children under 5 years of age, particularly in developing countries (1). Although most of these infections are mild and self-limiting within a few days in immunocompetent children in developed countries, certain populations, such as older adults, young children, and individuals with chronic illnesses or weakened immune systems may experience significant morbidity requiring medical intervention (2–6). These diseases are caused by a variety of pathogens, including bacteria, viruses, and parasites. Determining the etiology of these gastrointestinal (GI) infections is essential to administer the appropriate treatment and take the proper infection control and public health measures (2, 7). Traditionally, the diagnosis of GI infections has relied on conventional methods, such as bacterial stool cultures and stool microscopy for ova and parasite detection. Despite their widespread use, these methods remain labor-intensive, costly, and require longer processing times (8, 9). In recent years, multiplex polymerase chain reaction (PCR)-based GI pathogen panels have been increasingly adopted to address the limitations of conventional approaches, enabling faster and accurate diagnosis for multiple enteropathogens simultaneously (8, 10). Several studies have indicated that multiplex PCR panels can improve clinical management and contribute to cost reductions by decreasing unnecessary antibiotic use, additional diagnostic testing, and length of hospital stay (11–13). However, studies comparing the diagnostic performance of multiplex PCR gastrointestinal panels with conventional methods remain limited, especially in pediatric populations (14, 15). The QIAGEN QIAstat-Dx Gastrointestinal Panel (QGP; Hilden, Germany) is a fully automated multiplex real-time PCR system that provides both qualitative results and quantitative cycle threshold (Ct) values for 23 gastrointestinal pathogens (16). This study aimed to evaluate the diagnostic yield, agreement, and turnaround time of the QIAstat-Dx Gastrointestinal Panel compared with stool culture and ova and parasite examination in a large pediatric population.

## MATERIALS AND METHODS

### Study design and setting

This retrospective study was conducted at Sidra Medicine, a pediatric tertiary care center in Qatar that provides inpatient, outpatient, and emergency services. The study included stool samples from patients aged ≤18 years who were being assessed for gastroenteritis and sent to the microbiology laboratory for routine analysis between 1 November 2022 and 30 September 2025. The samples were tested immediately upon receipt using one or more of the following methods: QIAstat-Dx GI Panel (*n* = 4,801), stool culture (*n* = 2,018), and ova and parasites (O&P) examination (*n* = 986). Patients hospitalized for over 72 h before collecting stool culture or 96 h before O&P testing were excluded from the study. Patient demographic data were obtained retrospectively through review of the electronic medical records.

### Diagnostic tests

#### QIAstat-Dx GI panel assay

The QIAstat-Dx gastrointestinal panel (QGP; QIAGEN, Hilden, Germany) is a molecular assay designed to qualitatively detect nucleic acids from 23 bacterial, viral, and parasitic pathogens that are commonly associated with gastroenteritis. In our study, we utilized version 2 of the QGP assay, which operates with the QIAstat-Dx Analyzer (QIAGEN, Hilden, Germany), an automated device that combines nucleic acid extraction with multiplex real-time PCR detection. This panel can identify a broad range of pathogens, including *Clostridium difficile* toxin A/B*,* enteroaggregative *E. coli* (EAEC), enteroinvasive *E. coli* (EIEC)/*Shigella,* enteropathogenic *E. coli* (EPEC), enterotoxigenic *E. coli* (ETEC), *Campylobacter* spp., *Plesiomonas shigelloides, Salmonella* spp., Shiga-like toxin-producing *E. coli* (STEC) *stx1/stx2, E. coli O157:H7, Vibrio cholerae, Vibrio parahaemolyticus, Vibrio vulnificus, Yersinia enterocolitica,* adenovirus, astrovirus, norovirus GI/GII, rotavirus A, sapovirus spp., *Cyclospora cayetanensis, Entamoeba histolytica, Cryptosporidium,* and *Giardia lamblia*. The assay was performed according to the manufacturer’s instructions. In brief, the stool samples were transferred to Cary–Blair medium prior to testing. Then, 200 μL of the diluted sample was vortexed and placed into the QGP test cartridge, which was sealed and inserted into the analyzer. The test takes 76 min, after which the software generates a comprehensive report detailing the results for all 23 pathogens and the internal control. For samples that tested positive, the PCR cycle threshold (Ct) value and amplification curves were also included in the report.

#### Stool culture

Stool cultures included routine screening to identify *Salmonella* spp.*, Shigella* spp.*, Campylobacter jejuni, Campylobacter coli, Vibrio* spp.*, E. coli O157:H7, Aeromonas* spp.*, Plesiomonas* spp., and *Y. enterocolitica*. The samples were inoculated into eight different culture medium plates: MacConkey agar, Columbia agar with 5% sheep blood, *Campylobacter* blood agar, Cefsulodin-Irgasan-Novobiocin (CIN) agar, Thiosulfate-Citrate-Bile-Sucrose (TCBS) agar, Sorbitol MacConkey (SMAC) agar, and Hektoen Enteric (HE) agar, and Selenite F enrichment broth (SEB) (Oxoid Limited, Basingstoke, United Kingdom). All plates were incubated for 72 h at 35°C in an O_2_ incubator, except *Campylobacter* blood agar plates, which were incubated at 42°C under microaerophilic conditions, and CIN plates, which were incubated at room temperature. All potential stool pathogens were identified at the genus level using matrix-assisted laser desorption/ionization time-of-flight mass spectrometry (MALDI-TOF MS) (Bruker, Bremen, Germany). Because MALDI-TOF MS does not reliably distinguish *Shigella* spp. from *E. coli*, presumptive isolates were further identified using the VITEK 2 Compact system (bioMérieux, Marcy-l’Étoile, France), followed by serological serotyping with the Wellcolex Color *Shigella* kit (Remel Europe Limited, Kent, United Kingdom), according to the manufacturers’ instructions. Serological serotyping was performed on all *Salmonella* spp. isolates using BD Difco *Salmonella* Vi Antiserum (Becton, Dickinson and Company, Maryland, USA) and Wellcolex Color *Salmonella* kit (Remel Europe Limited, Kent, United Kingdom), following the instructions in the package insert.

#### Ova and parasite

The stool samples were examined using a saline wet mount, concentrated using Mini Parasep Solvent Free (Apacor Limited, Berkshire, United Kingdom), and stained with trichrome stain (Atom Scientific, Hyde, United Kingdom). Additionally, the Merifluor Cryptosporidium/Giardia kit (Cincinnati, Ohio, United States, Meridian Bioscience) direct fluorescent assay was used to detect *Cryptosporidium* spp. and *G. lamblia*. A modified acid-fast stain (Prolab Diagnostics, Neston, United Kingdom) was performed to confirm positive *Cryptosporidium* results detected by the Merifluor Cryptosporidium/Giardia kit or when *Cyclospora* infection was suspected or specifically requested by the physician. All tests were performed according to the manufacturer’s instructions.

### Statistical analysis

The assessment of the QGP performance involved comparing it with traditional tests (stool culture and ova and parasite), which served as the reference methods. For each target identified by the QGP, false positives (FPs), true positives (TPs), true negatives (TNs), and false negatives (FNs) were assigned. Positive percent agreement (PPA) and negative percent agreement (NPA) were calculated with 95% confidence intervals (CIs). Stool culture and ova and parasite served as the reference methods. As this was a retrospective study, no predefined pairing criteria were used. Test selection was clinician-directed, and pairing occurred only when multiple assays were ordered for the same specimen on the same day. For paired samples, agreement was assessed using the Kappa coefficient, and McNemar’s test was applied, with a *P*-value of ≤0.05 indicating a statistically significant difference. All analyses were performed using MedCalc’s Diagnostic Test Calculator (https://www.medcalc.org/en/calc/diagnostic_test.php). The positivity rate and the median turnaround time for each test were also calculated.

## RESULTS

### Patient demographics and diagnostic testing methods

From 1 November 2022 to 30 September 2025, 4,801 samples were processed using the QIAstat-Dx QGP, 2,018 samples were subjected to stool culture analysis, and 986 samples were tested for ova and parasites. Among the QGP sample, 1,448 (72%) were ordered concurrently with the stool culture, and 663 (67%) were ordered with the ova and parasite samples. The median age of the patients was 4 years (interquartile range 1–10), with an average age of 5.6 years (SD = 5), ranging from 0 to 18 years. Most patients (57%) were younger than 6 years, 31% were between 6 and 12 years, and 12% were aged 13–18 years. Of the samples collected, 2,723 (57%) were from male patients, while 2,078 (43%) were from female patients. Half of the samples, 2,400 (50%), were obtained from inpatient units, 960 (20%) from outpatient clinics, and 1,441 (30%) from the emergency department. The patient demographics for the overall study population as well as for each cohort (QGP, stool culture, and O&P) are illustrated in Table 1.

**TABLE 1:** Demographic and clinical characteristics of the study population^*a*^

Characteristic	Overall populations	QGP (*n* = 4,801)	Stool culture (*n* = 2,018)	O&P (*n* = 986)
Age (years)				
Median (IQR)	4 (1–10)	4 (1–10)	5 (2–10)	7 (3–11)
Avg (SD)	5.7 (5)	5.6 (5)	6.2 (5)	7.1 (5)
Age group (%)				
<6 years	57	56	52	43
6–12 years	31	32	34	40
13–18 years	12	12	14	17
Gender (%)				
Male	57	56	55	55
Female	43	44	45	45
Patient location (%)				
Emergency department	30	29	36	22
Inpatient	50	53	34	36
Outpatient	20	18	30	42

^*a*^ IQR, interquartile range; *n*, number; QGP, QIAstat-Dx gastrointestinal panel; SD, standard deviation.

### Performance characteristics of QGP

The positivity rates for various pathogen groups detected by QGP and conventional methods, along with the median time required to complete each test, are detailed in Table 2. Out of the 4,801 samples tested by QGP, 2,033 (42.3%) were positive. A single pathogen was detected in 1,448 (30.1%) samples, and two or more pathogens were detected in 585 (12.2%) samples. A total of 2,814 pathogens were identified in the 2,033 positive samples; EPEC was the most prevalent, found in 631 samples (13.1%), followed by EAEC in 380 samples (7.9%). Norovirus GI/GII was the most frequently identified virus, detected in 354 samples (7.3%). *Cryptosporidium* spp. was the most common parasite, detected in 79 samples (1.6%), followed by *G. lamblia* in 56 (1.1%). *V. vulnificus* and *E. histolytica* were not detected in any samples. The QGP also demonstrated a rapid turnaround time, with a median of 2.8 h in comparison to stool culture (67.6 h) and O&P examination (38.8 h).

**TABLE 2:** Median and mean (95% CI) turnaround time and positivity rates of various pathogen groups detected by QGP, stool culture, and O&P examination^*a*^

Pathogen	Positive, *n* (%)		
	QGP (Total samples tested, *n =* 4,801)	Stool culture (Total samples tested, *n =* 2,018)	O&P (Total samples tested, *n* = 986)
Bacterial group			
Total positive bacteria	1780 (37)	255 (12.6)	NA
EAEC	380 (7.9)	NA	NA
EPEC	631 (13.1)	NA	NA
EIEC	67 (1.4)	NA	NA
ETEC	99 (2.0)	NA	NA
STEC	102 (2.1)	NA	NA
*Campylobacter* species	154 (3.2)	63 (3.1)	NA
*Salmonella* species	212 (4.4)	134 (6.6)	NA
*E. coli* O157	33 (0.6)	1 (0.04)	NA
*Plesiomonas shigelloides*	7 (0.14)	0 (0)	NA
*Yersinia enterocolitica*	5 (0.10)	1 (0.04)	NA
*Vibrio cholerae*	6 (0.12)	1 (0.04)	NA
*Vibrio parahaemolyticus*	1 (0.02)	0 (0)	NA
*Vibrio vulnificus*	0 (0)	0 (0)	NA
*C. difficile*	83 (1.7)	NA	NA
*Aeromonas* species	NA	46 (2.3)	NA
*Shigella* species	NA	9 (0.4)	NA
Virus group			
Total positive viruses	897 (18.6)	NA	NA
Norovirus GI/GII	354 (7.3)	NA	NA
Rotavirus	209 (4.3)	NA	NA
Adenovirus	80 (1.6)	NA	NA
Sapovirus	168 (3.4)	NA	NA
Astrovirus	86 (1.8)	NA	NA
Parasite group			
Total positive parasites	137 (2.8)	NA	63 (6.3)
*Cryptosporidium* species	79 (1.6)	NA	15 (1.5)
*Giardia lamblia*	56 (1.1)	NA	24 (2.4)
*Cyclospora cayetanensis*	2 (0.04)	NA	NA
*Entamoeba histolytica*	0 (0)	NA	0 (0)
*Blastocystis hominis*	NA	NA	22 (2.2)
*Hymenolepis nana*	NA	NA	2 (0.2)
Overall positive	2,033 (42.3)	255 (12.6)	63 (6.3)
Median TAT (h)	2.81	67.6	38.8
Mean TAT, h (95% CI)	5.93 (2.33–9.52)	69.97 (69.40–70.53)	38.39 (37.18–39.61)

^*a*^ EAEC*, *enteroaggregative* E. coli*; EPEC*, *enteropathogenic* E. coli*; ETEC, enterotoxigenic* E. coli*; EIEC, enterinvasive *E. coli*; QGP, QIAstat-Dx gastrointestinal panel; STEC, Shiga-like toxin-producing* E. coli*; TAT, turnaround time; NA, not applicable.

Analysis of the stool culture samples showed that 255 (12.6%) pathogens were detected in 2,018 samples. *Salmonella* spp. was the most prevalent, detected in 134 samples (6.6%), followed by *Campylobacter* spp. in 63 samples (3.1%), and *Aeromonas* spp. in 46 samples (2.3%). Among the 986 samples tested for O&P, the overall positivity rate was 63/986 (6.3%). The most frequently detected parasites were *G. lamblia* in 24 samples (2.4%) and *Cryptosporidium* in 15 samples (1.5%).

### Agreement between QGP and stool culture

**Fig 1:**
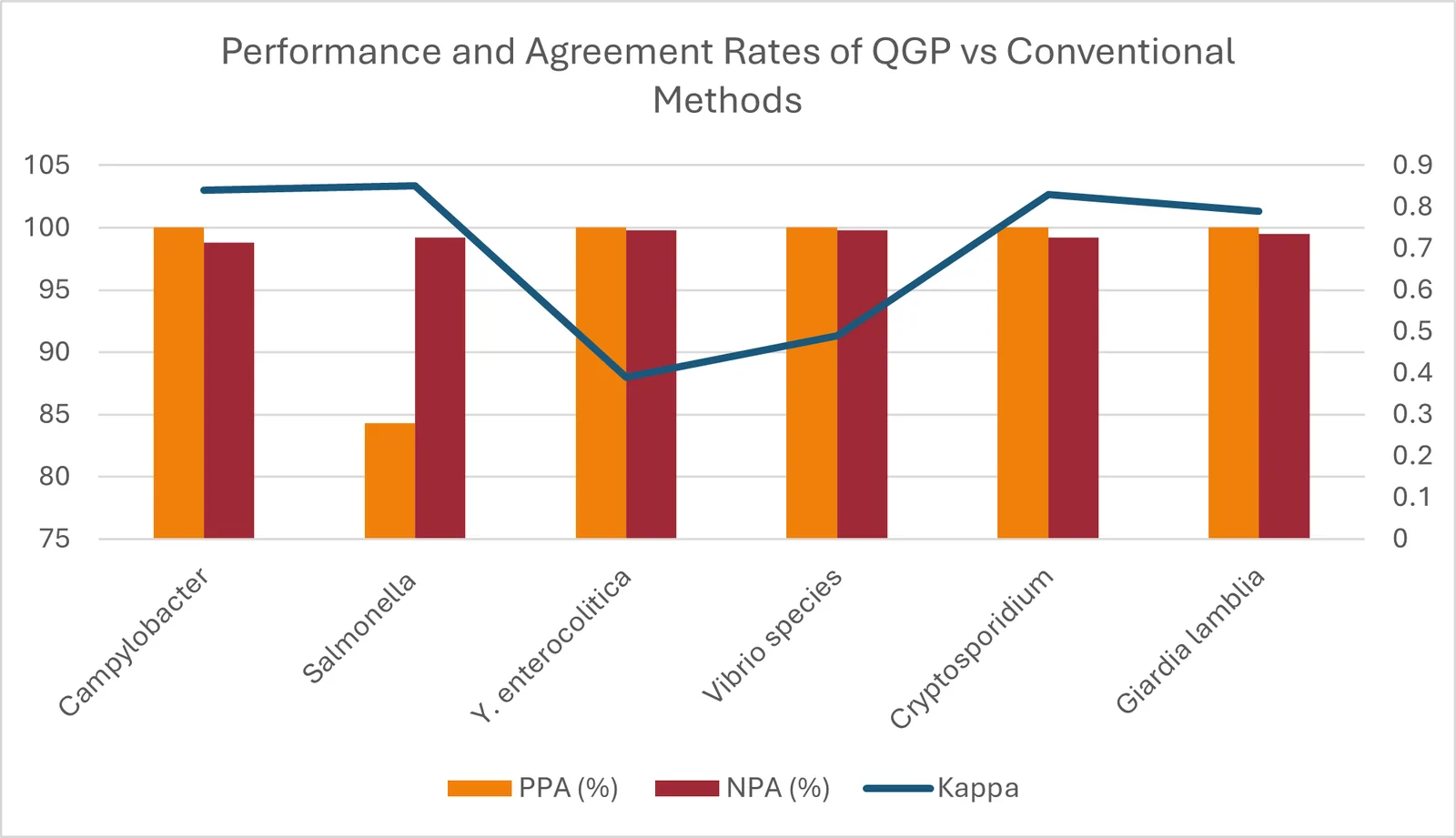
Performance and agreement rates for QGP in comparison to conventional methods across different pathogens. Abbreviations: NPA, negative percent agreement; PPA, positive percent agreement; QGP, QIAstat-Dx gastrointestinal panel.

Stool culture and QGP were simultaneously requested for 1,448 patients. The PPA, NPA, kappa coefficients, and *P* values for each target tested by the QGP and stool culture are listed in Table 3. The agreement rates of the QGP and stool culture are shown in Fig. 1. The PPA and NPA were 89.6% and 96.2%, respectively. The kappa coefficient was 0.77, with a *P*-value of <0.0001, indicating good agreement. For *Campylobacter,* the agreement was exceptionally high, with a PPA of 100% and NPA of 98.8%. The kappa coefficient was 0.84, and the *P*-value was <0.0001. However, for *Salmonella* spp., despite a kappa coefficient of 0.85 and NPA of 99.2%, the PPA was lower at 84.4%, with a *P*-value of 0.5. *Y. enterocolitica* and *Vibrio* spp. had a PPA of 100%. *E. coli* O157 and *P. shigelloides* were detected only by the QGP. The QGP detected co-infections with two or more bacterial pathogens that were also included in routine stool culture testing in seven out of eight samples (failed to detect *Salmonella* spp. in one sample). In contrast, stool culture identified only two co-infections involving *Salmonella* spp. and *Campylobacter* spp. Culture failed to detect *Campylobacter* spp. in three samples and *E. coli* O157 in the remaining three samples.

**TABLE 3:** Performance and agreement rates of QGP vs conventional methods^*a*^

Pathogens	TP	FN	FP	TN	PPA (%)	NPA (%)	Kappa coefficient (95% CI)	*P* value
Overall agreement between QGP and stool culture (*n* = 1448)^*c*^	130	15	49	1,254	89.6	96.2	0.77 (0.72–0.83)	<0.0001
*Campylobacter* species	47	0	17	1,384	100	98.8	0.84 (0.76–0.91)	<0.0001
*Salmonella* species	81	15	11	1,341	84.4	99.2	0.85 (0.79–0.90)	0.5
*E. coli* O157	0	0	12	1,436	–	99.2	–^*b*^	0.0005
*Plesiomonas shigelloides*	0	0	4	1,444	–	99.7	–	0.12
*Yersinia enterocolitica*	1	0	3	1,444	100	99.8	0.39 (0.14–0.94)	0.25
*Vibrio* species	1	0	2	1,445	100	99.9	0.49 (0.1–1.0)	0.5
Overall agreement between QGP and ova & parasite (*n* = 663)^*c*^	19	0	8	636	100	98.7	0.82 (0.69–0.94)	0.007
*Cryptosporidium* species	13	0	5	645	100	99.2	0.83 (0.69–0.97)	0.06
*Giardia lamblia*	6	0	3	654	100	99.5	0.79 (0.57%–1%)	0.25
*Entamoeba histolytica*	0	0	0	663	–	–	–	–

^*a*^ FN, false negative; FP, false positive; *n*, number; NPA, negative percent agreement; PPA, positive percent agreement; QGP, QIAstat-Dx gastrointestinal panel; TN, true negative; TP, true positive.

^*b*^ “–” indicates that the value could not be calculated because of a lack of positive samples.

^*c*^ Grey shading indicates overall agreement between methods.

### Agreement between QGP and ova and parasite examination

Stool culture and O&P were simultaneously requested for 663 patients. The PPA, NPA, kappa coefficients, and *P* values for each target tested by the QGP and O&P are listed in Table 3. The agreement rates of the QGP and O&P are shown in Fig. 1. The PPA and NPA were 100% and 98.7%, respectively. The kappa coefficient was 0.82, with a *P-*value of 0.007. Both *Cryptosporidium* spp. and *G. lamblia* achieved a PPA of 100%, with NPA and kappa values of 99.2% (0.83) and 99.5% (0.79), respectively. One sample had a co-infection with both *Cryptosporidium* spp. and *G. lamblia*, which was detected by both methods.

## DISCUSSION

We evaluated the diagnostic performance of the QGP in our pediatric population in comparison with conventional stool culture and ova and parasite examination. Our study showed a high level of agreement between QGP with conventional diagnostic methods, achieving more than 98% NPA and 100% PPA for all targets except *Salmonella* spp. (100% NPA, 84.4% PPA). A previous study evaluating QGP reported PPA and NPA of 34.8% and 85.3%, respectively, compared with stool culture, and 0.0% and 78.3%, respectively, compared with microscopic examination (15). Among the 125 positive samples evaluated in that study, 13 discrepant results were observed, including five *Campylobacter* spp., one *Salmonella* spp., and four *G. lamblia* detected by QGP but negative by culture or microscopy, likely reflecting the lower sensitivity of conventional diagnostic methods (15).

The lower PPA for *Salmonella* spp. in our study can be explained by the 15 isolates that grew only from culture upon incubation in SEB. This suggests that these isolates were likely present at low bacterial loads in the stool sample, below the QGP detection threshold. According to previous studies, SEB can improve the detection of *Salmonella* spp. by three-fold in comparison to direct plating, and it can enhance *Salmonella* spp. detection by 13.2% using SEB with fecal DNA extraction to improve the sensitivity of molecular diagnostics for *Salmonella spp* (17, 18). Similar to our findings, a previous study evaluating the Luminex xTAG Gastrointestinal Pathogen Panel reported PPA values exceeding 87% for most bacterial targets; however, agreement for *Salmonella* spp. was lower, with a PPA of 78.2%, and 12 of 51 positive cases were detected solely by culture (19).

The use of QGP testing increased the detection rates of gastrointestinal pathogens compared with conventional stool culture and ova and parasite (O&P) examination. In our study, QGP identified one or more pathogens in 42.3% of the submitted samples, exceeding the detection rates of stool culture (12.6%) and O&P testing (6.3%). These findings concur with studies conducted in both pediatric and adult patients, where multiple PCR assays identified a higher number of pathogens compared with conventional methods (14, 15, 20–23). Although data on children are limited, one previous pediatric study reported a pathogen detection rate of 68.8% using QGP, compared with 35.2% using conventional methods (15). Similarly, Aydemir et al. (14) found a positivity rate of 51.8% in both pediatric and adult populations, outperforming traditional diagnostic techniques (14). Both studies revealed that certain pathogens identified by QGP were not detected using conventional methods including rapid antigen tests for rotavirus and adenovirus (14, 15).

Gastrointestinal coinfections are more commonly observed in pediatric patients (14). Commercially available multiplex molecular panels, although differing in the number of detectable targets, consistently demonstrate a superior ability to identify mixed coinfections in comparison with traditional methods (24, 25). In our study, coinfections were detected in a in 28.4% of QGP-positive samples. This finding is consistent with multiple previous studies utilizing multiplex PCR assays, where coinfection rates ranged from 12% to 42% (14, 15, 24, 26). In addition, QGP detected six additional bacterial co-infections that were not identified by routine stool culture. This highlights the robust performance of QGP in detecting multiple gastrointestinal pathogens within the same specimen. Some bacterial pathogens, such as *Campylobacter* spp. and *E. coli* O157, can be difficult to recover by conventional stool culture because they require specialized media and organism-specific growth conditions. These factors may limit the ability of stool culture to detect co-infections. Previous studies have reported that *Campylobacter* spp. may fail to grow in up to 30% of stool cultures, while *E. coli* O157 can be difficult to isolate using routine stool culture due to the lack of distinctive phenotypic characteristics that differentiate it from non-pathogenic *E. coli* strains (27, 28). Although molecular panels improve sensitivity and the detection of co-infections, they may also detect nucleic acids from nonviable organisms or asymptomatic colonization, potentially overestimating clinically relevant co-infections (14). Therefore, results should be interpreted cautiously in the context of the patient’s clinical presentation.

In our study, the most common pathogens were EPEC (631 cases), EAEC (380 cases), and Norovirus GI/GII (354 cases). These pathogens are among the top six causes of gastrointestinal infections and have been identified by other researchers in the United States and Europe using multiplex PCR panel-based testing methods (26, 29). EPEC and EAEC infections occur more frequently in children than in adults and are common pathogens, particularly in urban areas of developed countries (30). For example, EPEC accounts for 29.5% of pathogens identified in a U.S. study, while it has been detected in 5%–10% of children with diarrhea in developing countries (26, 31). Norovirus, the third most prevalent pathogen in this study, is associated with approximately 18% of acute gastroenteritis worldwide and one million healthcare visits in the Unnited States, with higher incidence rates in children under 5 years and adults over 65 years (32, 33).

Our study demonstrated that QGP significantly reduced the turnaround time from sample collection to result to 2.8 h, compared with an average of 67.6 h for stool culture and 38.8 h for O&P testing, which is consistent with previous studies using multiplex PCR assays (34, 35). This reduced reporting time can lead to quicker decisions in patient management and implementing infection control precautions.

Despite all the advantages mentioned above, molecular assays cannot fully replace culture due to their inability to determine antimicrobial susceptibility testing, which is recommended in certain risk groups (36, 37). In addition, molecular panels do not include all potential enteric pathogens, such as *Aeromonas* spp., *Blastocystis hominis*, and *Hymenolepis nana*, which rarely cause diarrheal disease in populations other than immunocompromised patients (38–40). Moreover, the panel does not differentiate between certain bacterial species, which is crucial for diagnosing specific pathogens, such as *Salmonella* spp., where distinguishing *Salmonella typhi* is clinically important (41). Another limitation is that molecular assays are highly sensitive detecting very low numbers of enteric pathogens and cannot differentiate between active infection and colonization status, posing a result interpretation challenge for pathogens such as *Clostridioides difficile* (37). However, several studies indicated that the use of PCR cycle threshold (Ct) values for positive samples can help in test interpretation and to differentiate between infection and colonization (42–44). Furthermore, a major limitation of this study is the absence of a secondary diagnostic method to confirm positive PCR results, which limited our ability to formally assess the sensitivity and specificity of the QGP assay compared with conventional methods.

### Conclusion

Multiplex molecular panels represent an advancement in diagnosing pediatric gastroenteritis, serving as a more sensitive, faster, and cost-effective alternative to traditional microbiological testing. Additional studies evaluating the clinical benefit of broadening pathogen detection, impact on clinical decision-making, and outcome improvement, compared with conventional methods are needed.

## Data Availability

The data sets generated and/or analyzed during the current study are available from the corresponding author on reasonable request.
